# Predictors of participant retention in a community-based HIV prevention cohort: perspectives from the HPTN 071 (PopART) study

**DOI:** 10.1186/s13063-023-07404-y

**Published:** 2023-06-27

**Authors:** Nomtha Bell-Mandla, Ethan Wilson, Deeksha Sharma, Rosa Sloot, Justin Bwalya, Ab Schaap, Deborah Donnell, Estelle Piwowar-Manning, Sian Floyd, Nozizwe Makola, Lungiswa Nkonki, Musonda Simwinga, Ayana Moore, Richard Hayes, Sarah Fidler, Helen Ayles, Peter Bock, the HPTN 071 (PopART) Study Team

**Affiliations:** 1 Desmond Tutu TB Centre, Department of Paediatrics and Child Health, Stellenbosch University, Cape Town, South Africa; 2 Fred Hutchinson Cancer Research Center, Seattle, WA USA; 3 Zambia AIDS Related Tuberculosis Project, University of Zambia, Lusaka, Zambia; 4 Department of Infectious Disease Epidemiology, London School of Hygiene and Tropical Medicine, London, UK; 5 Johns Hopkins University School of Medicine, Baltimore, USA; 6 Department of Global Health, Division of Health Systems and Public Health, Faculty of Medicine and Health Sciences, Stellenbosch University, Cape Town, South Africa; 7 FHI360, Durham, NC FHI360 USA; 8 Department of Infectious Disease Epidemiology, Imperial College London, London, UK; 9 Department of Clinical Research, London School of Hygiene and Tropical Medicine, London, UK

**Keywords:** Community randomized trial, HIV, Longitudinal research, Retention, Gender, Age, Study precision

## Abstract

**Introduction:**

In 2021, there were 38.4 million people living with HIV (PLHIV) globally, of which 20.6 million (54%) were living in Eastern and Southern Africa. Longitudinal studies, inclusive of community randomized trials (CRTs), provide critical evidence to guide a broad range of health care interventions including HIV prevention. In this study, we have used an individual-level cohort study design to evaluate the association between sex and other baseline characteristics and participant retention in the HPTN 071 (PopART) trial in Zambia and South Africa.

**Methods:**

HPTN 071 (PopART) was a community randomized trial (CRT) conducted from 2013 to 2018, in 21 communities. The primary outcome was measured in a randomly selected population cohort (PC), followed up over 3 to 4 years at annual rounds. PC retention was defined as completion of an annual follow-up questionnaire. Baseline characteristics were described by study arm and Poisson regression analyses used to measure the association between baseline factors and retention. In addition, we present a description of researcher-documented reasons for study withdrawal by PC participants.

**Results:**

Of the 38,474 participants enrolled during the first round of the trial (PC0), most were women (27,139, 71%) and 73% completed at least one follow-up visit. Retention was lower in men (adj RR: 0.90; 95% CI: 0.88, 0.91) and higher among older participants (adj RR: 1.23; 95% CI 1.20, 1.26) when comparing ages 35–44 to 18–24 years. Retention was higher among individuals with high socioeconomic status (SES) (adj RR 1.16; 95% CI 1.14, 1.19) and medium SES (adj RR 1.12; 95% CI 1.09, 1.14) compared to low SES. The most common reasons for study withdrawal were study refusal (23%) and relocation outside the CRT catchment area (66%).

**Conclusion:**

Despite challenges, satisfactory retention outcomes were achieved in PopART with limited variability across study arms. In keeping with other studies, younger age, male sex, and lower SES were associated with lower levels of retention. Relocation outside of catchment area was the most common reason for non-retention in this CRT.

## Introduction

In 2021, there were 38.4 million people living with HIV (PLHIV) globally, most of whom (20.6 million, 54%) were in Eastern and Southern Africa with 1.5 million new HIV infections globally [1]. There remains considerable scope to innovate and strengthen HIV prevention services in high burden settings. Longitudinal studies, inclusive of cohort studies, randomized controlled trials (RCTs), and community randomized trials (CRTs) provide critical evidence to guide a broad range of health care interventions including HIV prevention [2] Although longitudinal studies provide high-quality data measuring the temporal association between risk factors and outcomes, their successful implementation is complex [2]. Poor retention on study, which may be differential for particular participant characteristics or study arms, remains one of the most significant challenges [2].

Participant retention outcomes vary across different types of longitudinal studies. Very high rates of loss to follow-up are routinely reported in observational cohort studies, commonly conducted within public health settings. For example, antiretroviral treatment (ART) cohort studies measuring programmatic outcomes in Africa report attrition rates of up to 50% at 1 year [3, 4]. Study attrition in RCTs, conducted in highly regulated environments, often at established research clinics, is overall much lower with HIV prevention RCTs from South Africa reporting retention of > 80% even after 3–4 years of follow-up [5, 6]. Achieving high participant retention in CRTs, typically conducted in real world settings, includes many of the challenges experienced in programmatic cohort studies. Follow-up in CRTs is further complicated by the need for participants to remain within a specified geographical area, with relocation and mobility out of communities being the most cited reason for attrition. Trial design, including methods for evaluating the primary endpoint in CRTs, differs, with some conducting longitudinal follow-up of all community members with access to the study intervention and others (including PopART) randomly selecting a smaller number of community members for cohort enrolment and follow-up [7, 8].

Research into reasons for poor retention in longitudinal studies has focused primarily on cohort studies and RCTs with few data from CRTs. Factors influencing retention in longitudinal studies may be considered at the levels of participant characteristics and contextual factors. Participant characteristics associated with higher attrition include being male [9], younger individuals (< 35 years) [9–11], belonging to an ethnic minority, lower socioeconomic status (SES), not being married, presence of mental health and substance abuse issues, and high-risk sexual behavior [9, 11]. For successful retention, effective community engagement, recruitment of suitable participants, and/or specially targeted retention efforts are critical. Many studies include eligibility criteria promoting retention, such as a stated intention to remain on contraception thereby reducing the chance of pregnancy where pregnancy is an exclusion criterion during follow-up. However, in some instances, recruitment of participants at higher risk of attrition may be required, to include a representative study sample and achieve study statistical power. For example, men and youth are often challenging to retain but their inclusion is important in HIV prevention research. In the absence of accurate algorithms for cross-sectional estimation of HIV incidence, we are still reliant on longitudinal follow-up. In this context, intention to remain in the study community is a common inclusion criterion in CRTs; however, participant mobility due to migration for work and other socioeconomic reasons both within and out of study communities remains a major challenge [12].

There remains a paucity of conclusive data on factors contributing to and effective strategies toward retention in longitudinal studies, not least from CRTs in high burden settings. In this study, we aimed to evaluate the association between baseline characteristics and retention of research participants in the PopART population cohort (PC), implemented in urban and peri-urban communities in Zambia and South Africa.

## Methods

HPTN 071 (PopART) was a three-arm CRT conducted from 2013 through 2018, in 21 urban or peri-urban communities in Zambia and South Africa [13, 14]. The aim was to evaluate the impact of an HIV combination prevention intervention, including universal testing and treatment, on population-level HIV incidence. The intervention included a package of HIV prevention services, incorporating HIV testing, delivered at household level by a cadre of community workers, known as community HIV-care providers (CHiPs). The household intervention was combined with active linkage, for HIV treatment and other services at government healthcare facilities. Study arms were as follows: arm A—CHiPs and universal ART for all HIV-positive individuals; arm B—CHiPs and ART as per local standard of care; and arm C—standard care. From October 2016, ART for all HIV-positive people became standard care and arms A and B were similar from that stage of study implementation. A study community was defined by the catchment population of a government clinic. Communities were grouped in triplets based on geographic location and estimated HIV prevalence. Each community in each triplet was randomly allocated to a separate arm of the study at simultaneous public ceremonies in Zambia and South Africa. The PopART primary outcome was measured in a population-based cohort (PC), implemented in all study communities regardless of study arm. Approximately 2000 participants were recruited in each community for the PC with 38,474 participants enrolled at study baseline (PC0) and followed up annually three times, at PC12 (12 months), PC24 (24 months), and PC36 (36 months). Additional participants were enrolled at PC12 and PC24 to increase sample size for primary outcome assessment. The primary outcome, measured between PC12 and PC36, reported in 2019, showed an overall 20% reduction in HIV incidence in the intervention arms (A and B) compared with standard care (arm C) [12].

PC participants were recruited according to the following steps: (i) household census data were collected prior to PC0 to create a sampling frame; (ii) households in trial communities were randomly selected; (iii) research enumerators approached each selected household for enumeration of individual residents in the household using an electronic data capture device (EDC); (iv) from each enumerated household, one adult aged 18–44 years was randomly selected for invitation to participate in the PC, following eligibility determination; (v) if the randomly selected individual was willing to participate, an informed consent process was conducted. PC inclusion criteria included aged 18–44 years and the intention to remain in the study community for the trial duration. In contrast to facility-based studies, PopART follow-up visits were conducted in households and communities with extensive retention-focused activities. Scheduled retention activities included flexible household visit schedules in the form of household visits completed during weekday evenings and Saturdays, to reach individuals who were engaged in work or educational activities during the day [15]. Repeated follow-up visits were made to the household when the participant was not found initially. In addition, small tokens of appreciation were issued to participants upon completion of study activities. Participant-researcher relationships were established and sustained during the course of the study and the dissemination period [15]. Extensive community engagement, involving communities and other key stakeholders (health services, community organizations, police services, etc.) in study activities right from the start, prior to random allocation of communities to study arms and throughout study implementation, was a key focus during PopART [16]. At each study visit, the research enumerator completed paper-based source documents and a study survey on the EDC. A research nurse collected a venous blood specimen for testing at the laboratory and offered a point of care HIV test.

The primary objective of the current study was to evaluate characteristics associated with follow-up retention among participants enrolled at PC0. Our primary retention outcome was defined as the total number of annual visits where a participant completed a study questionnaire, with values ranging from 0 to 3. A secondary aim was to characterize retention during the study, including visit-level summaries of incomplete study visit outcomes (missed and withdrawal visits), as well as reasons for study withdrawal.

PC survey data and laboratory outcomes were used in this analysis. Only data from participants enrolled at PC0 were included; participants enrolled at PC12 and PC24 were excluded from the analysis, to simplify the presentation and interpretation of results. A standardized electronic questionnaire (survey), completed at each annual round, included questions on demographic, socioeconomic, and biomedical factors. Questionnaires were administered by research enumerators who entered responses directly into the EDC which was programmed to include automated quality control steps. EDC data were uploaded in real time to the PC database managed by the HPTN data and statistical monitoring center (SCHARP). Data from laboratory test results were subsequently linked to questionnaire data in the database at SCHARP. Extensive iterative quality assurance processes were led by SCHARP with regular communication with site colleagues for resolution.

Baseline variables providing a description of the research sample and hypothesized a priori to be related to retention, where data were available, were included in the analysis. Variable categories were chosen to align with those used for PopART primary and secondary analyses. We investigated the following variables: age (18–24, 25–34, 35–44); sex (male, female); marital status (married/living as married, never married, divorced/separated/widowed); nights spent away from home in the past 3 months (none, 1 to 7, 8 to 30, > 30); socioeconomic factors—education level (none to primary, partial or full secondary school, college/university), employment status (employed, student, unemployed), country-specific socioeconomic status (SES) level (low, medium, high, calculated using methods described in the supplementary materials); study factors—arm (arm A, arm B, arm C); behavioral factors—multiple sex partners in the past 12 months (yes, no), condom-less last sex (yes, no), alcohol use disorder identification test (AUDIT) score (categories low: 0–7 medium: 8–14, high: 15–40) [17], recreational drug use (yes, no); health care factors—lab-confirmed baseline HIV status, sick days taken off from work in past 3 months (none, 1–5, > 5), disability status, self-reported HIV status and care (HIV-negative, don’t know, never tested, HIV-positive and have registered for HIV care, HIV-positive and have never registered for HIV care), self-report of ever told you have tuberculosis (yes, no). Number and percent of baseline characteristics were summarized among all participants enrolled in PC0, by arm and overall.

Combinations of longitudinal patterns of retention at the participant level were described in terms of retained visits (where a participant completed a study questionnaire) and not retained visits. Not retained visits were classified as either “missed visits” where follow-up was attempted at the next round or “withdrawal visits” where the participant was exited from the study. For each follow-up visit (PC12, PC24, PC36), we summarized the proportion of PC0 enrolled participants that were retained or had a missed or withdrawal visit, as well as reasons for study withdrawal. At PC36, all participants were considered retained or not retained, since it was the final study visit.

We modeled retained visits using univariable and multivariable Poisson regression with robust Huber-White standard error estimates. All participants were assumed to have the same rate denominator of 3 possible follow-up visits. Our final multivariable model included sex as the primary exposure of interest. The following variables were hypothesized a priori to be related to retention: age, education, marital status, employment, country-specific SES, AUDIT score, and lab-confirmed HIV status. We additionally included the remaining variables (arm, nights spent away from home in the past 3 months, multiple sex partners in the past 12 months, condom-less last sex, recreational drug use, sick days taken off from work in past 3 months, disability status, self-reported HIV status and care, and self-report of ever told you have tuberculosis) if they showed a significant association (*p* < 0.05) in univariable analyses.

Ethical approval of the HPTN 071 (PopART) trial protocol was obtained from the ethics committees of the University of Zambia (HPTN071/PopART UNZA BREC REF: 011–11-12), Stellenbosch University (N12/11/074), and the London School of Hygiene and Tropical Medicine (6326). All participants included in this study have undergone a full informed consent process for PopART.

## Results

Overall, 38,474 individuals aged 18–44 years were enrolled at PC0, with 12,671 (33%) in arm A, 13,404 (35%) in arm B, and 12,399 (32%) in arm C. The majority of participants were women 27,139 (71%). Overall, 15,225 (40%) were aged 18–24 years, 14,786 (39%) 25–34 years, and 8325 (22%) 35–44 years (Table 1). Age and sex distributions were similar across study arms. Most participants, 28,594 (76%), had secondary school education, with a small number, 2167 (6%), having attended College/University, 15,266 (40%) were married or living as married, and 23,625 (62%) were unemployed, with proportions similar across study arms. The proportion of participants classified as low, medium, or high SES were somewhat disparate by arm, with more participants reporting medium or high SES in arm C (78%) compared to arms A (61%) and B (59%). Few participants, 2243 (6%), reported having multiple sex partners in the past 12 months, and this was similar across arms. A high proportion (41%) reported that their last sex act was unprotected and this was highest in arm A (46%) followed by arms C (40%) and B (37%). The majority of participants (90%) were classified as low risk for alcohol abuse using the AUDIT scale. Medium to high alcohol risk was slightly higher in arms B and C. Overall, reported recreational drug use was low (1159, 3%) and similar across arms.

**Table 1:** Baseline characteristics at PC0

	**Total**	**Arm A**	**Arm B**	**Arm C**
Country				
South Africa	18,750/38,474 (49%)	6171/12,671 (49%)	6971/13,404 (52%)	5608/12,399 (45%)
Zambia	19,724/38,474 (51%)	6500/12,671 (51%)	6433/13,404 (48%)	6791/12,399 (55%)
Age				
18–24	15,225/38,336 (40%)	5065/12,636 (40%)	5179/13,364 (39%)	4981/12,336 (40%)
25–34	14,786/38,336 (39%)	4928/12,636 (39%)	5170/13,364 (39%)	4688/12,336 (38%)
35 +	8325/38,336 (22%)	2643/12,636 (21%)	3015/13,364 (23%)	2667/12,336 (22%)
Sex				
Male	11,202/38,341 (29%)	3595/12,637 (28%)	3906/13,364 (29%)	3701/12,340 (30%)
Female	27,139/38,341 (71%)	9042/12,637 (72%)	9458/13,364 (71%)	8639/12,340 (70%)
Education				
None to primary	6989/37,750 (19%)	2596/12,377 (21%)	2439/13,195 (18%)	1954/12,178 (16%)
Full to partial secondary school	28,594/37,750 (76%)	9070/12,377 (73%)	10,085/13,195 (76%)	9439/12,178 (78%)
College/university	2167/37,750 (6%)	711/12,377 (6%)	671/13,195 (5%)	785/12,178 (6%)
Marital status				
Married/living as married	15,266/37,992 (40%)	5363/12,560 (43%)	5210/13,233 (39%)	4693/12,199 (38%)
Never married	19,859/37,992 (52%)	6292/12,560 (50%)	6923/13,233 (52%)	6644/12,199 (54%)
Divorced/separated/widowed	2867/37,992 (8%)	905/12,560 (7%)	1100/13,233 (8%)	862/12,199 (7%)
Employment status				
Employed	9689/38,057 (25%)	3088/12,579 (25%)	3916/13,250 (30%)	2685/12,228 (22%)
Student	4743/38,057 (12%)	1568/12,579 (12%)	1394/13,250 (11%)	1781/12,228 (15%)
Unemployed	23,625/38,057 (62%)	7923/12,579 (63%)	7940/13,250 (60%)	7762/12,228 (63%)
Days off sick from work (past 3 mo.)				
None	32,826/36,178 (91%)	11,368/12,406 (92%)	11,052/12,151 (91%)	10,406/11,621 (90%)
1 to 5	1900/36,178 (5%)	603/12,406 (5%)	590/12,151 (5%)	707/11,621 (6%)
Greater than 5	1452/36,178 (4%)	435/12,406 (4%)	509/12,151 (4%)	508/11,621 (4%)
Nights away from home (past 3 mo.)				
None	32,224/36,806 (88%)	11,403/12,434 (92%)	10,304/12,190 (85%)	10,517/12,182 (86%)
1 to 7	3020/36,806 (8%)	762/12,434 (6%)	1117/12,190 (9%)	1141/12,182 (9%)
8 to30	1253/36,806 (3%)	209/12,434 (2%)	632/12,190 (5%)	412/12,182 (3%)
> 30	309/36,806 (1%)	60/12,434 (< 1%)	137/12,190 (1%)	112/12,182 (1%)
Country-specific SES				
Low	12,121/35,896 (34%)	4529/11,693 (39%)	5006/12,204 (41%)	2586/11,999 (22%)
Medium	11,847/35,896 (33%)	3511/11,693 (30%)	3503/12,204 (29%)	4833/11,999 (40%)
High	11,928/35,896 (33%)	3653/11,693 (31%)	3695/12,204 (30%)	4580/11,999 (38%)
Self-report HIV status and care				
Negative	17,823/36,060 (49%)	6514/11,752 (55%)	6592/12,539 (53%)	4717/11,769 (40%)
Never tested	5601/36,060 (16%)	1382/11,752 (12%)	2118/12,539 (17%)	2101/11,769 (18%)
Do not know	8481/36,060 (24%)	2603/11,752 (22%)	2275/12,539 (18%)	3603/11,769 (31%)
Positive, never registered for care	891/36,060 (2%)	286/11,752 (2%)	322/12,539 (3%)	283/11,769 (2%)
Positive, have registered for care	3264/36,060 (9%)	967/11,752 (8%)	1232/12,539 (10%)	1065/11,769 (9%)
Ever told you have tuberculosis				
Yes	511/38,006 (1%)	187/12,560 (1%)	128/13,222 (1%)	196/12,224 (2%)
No	37,495/38,006 (99%)	12,373/12,560 (99%)	13,094/13,222 (99%)	12,028/12,224 (98%)
Multiple sex partners (past 12 mo.)				
No	34,222/36,465 (94%)	11,192/11,758 (95%)	11,929/12,803 (93%)	11,101/11,904 (93%)
Yes	2243/36,465 (6%)	566/11,758 (5%)	874/12,803 (7%)	803/11,904 (7%)
Condom-less last sex				
No	21,573/36,642 (59%)	6519/12,069 (54%)	7982/12,765 (63%)	7072/11,808 (60%)
Yes	15,069/36,642 (41%)	5550/12,069 (46%)	4783/12,765 (37%)	4736/11,808 (40%)
AUDIT risk level				
Low risk	31,772/35,289 (90%)	10,738/11,541 (93%)	10,850/12,325 (88%)	10,184/11,423 (89%)
Medium risk	2476/35,289 (7%)	544/11,541 (5%)	1062/12,325 (9%)	870/11,423 (8%)
High risk	1041/35,289 (3%)	259/11,541 (2%)	413/12,325 (3%)	369/11,423 (3%)
Used drugs recreationally (past 12 mo.)				
No	36,729/37,888 (97%)	12,196/12,510 (97%)	12,722/13,168 (97%)	11,811/12,210 (97%)
Yes	1159/37,888 (3%)	314/12,510 (3%)	446/13,168 (3%)	399/12,210 (3%)
Baseline HIV status (lab confirmed)				
Negative	29,130/37,134 (78%)	9594/12,177 (79%)	10,235/12,969 (79%)	9301/11,988 (78%)
Positive	8004/37,134 (22%)	2583/12,177 (21%)	2734/12,969 (21%)	2687/11,988 (22%)

A total of 17,823 (49%) participants self-reported HIV-negative status at PC0; this proportion was higher in arms A and B compared to arm C. A further 16% and 24% reported “never tested” or “did not know” respectively. Overall, 891 (2%) reported “HIV positive never in care” and 3264 (9%) “HIV positive in care,” and these proportions were similar across arms. On laboratory testing, 78% and 22% of participants were HIV negative and HIV positive respectively with very similar proportions across study arms (Table 1).

Of the 38,474 participants enrolled at PC0, 72.6% completed at least one follow-up study visit and 10,526 (27.4%) had no follow-up visits completed (Table 2). The breakdown by number of follow-up visits completed was 16,916 (44.0%) were retained at all three follow-up visits, 5 609 (14.6%) two follow-up visits, and 5423 (14.1%) only 1 follow-up visit. On average participants completed 1.8 of 3 expected follow-up visits. Among those with mixed retention status (total of one or two retained visits during the study), incomplete visits were higher in the later rounds of follow-up (Table 2). Retention of eligible participants at each PC round was 66% at PC12, 65% at PC24, and 71% at PC36 (Table 3). Across all visits, study withdrawals accounted for 49% of non-retained visits, the most common reasons for which were refusal (23%) and relocation outside the CRT catchment area (66%). Other reasons included death (4%), incapacitation, or admission to hospital (2%) (Table 3).

**Table 2:** Retention rates and longitudinal retention patterns among PC0 enrolled

*Longitudinal retention pattern*^*a*^					
Total	*N* (%)	PC12	PC24	PC36	*N* (%)
3	16,916 (44.0%)	Retained	Retained	Retained	16,916 (44.0%)
		Retained	Retained	Not retained	2708 (7.0%)
2	5609 (14.6%)	Not retained	Retained	Retained	1503 (3.9%)
		Retained	Not retained	Retained	1398 (3.6%)
		Retained	Not retained	Not retained	4267 (11.1%)
1	5423 (14.1%)	Not retained	Not retained	Retained	605 (1.6%)
		Not retained	Retained	Not retained	551 (1.4%)
0	10,526 (27.4%)	Not retained	Not retained	Not retained	10,526 (27.4%)

^a^“Not retained” indicates either a missed visit, study withdrawal visit, or post-study withdrawal visit

**Table 3:** Visit-level retention and reasons for withdrawal among PC0 enrolled

	**PC12**	**PC24**	**PC36**	**Cumulative (all rounds)**
**Available for follow-up**	38,474	33,283	28,701	100,458
**Retained**	25,289 (65.7%)	21,678 (65.1%)	20,422 (71.2%)	67,389 (67.1%)
**Not retained**	13,185 (34.3%)	11,605 (34.9%)	8279 (28.8%)	33,069 (32.9%)
*Reasons for non-retention*				
**Missed visit**^**a**^	7994 (60.6%)	7023 (60.5%)	1799 (21.7%)	16,816 (50.9%)
**Terminated**	5191 (39.4%)	4582 (39.5%)	6480 (78.3%)	16,253 (49.1%)
*Reasons for withdrawal*				
**Death**	237 (4.6%)	181 (4%)	157 (2.4%)	575 (3.5%)
**Incapacitated/in hospital**	255 (4.9%)	6 (0.1%)	17 (0.3%)	278 (1.7%)
**Incarcerated**	26 (0.5%)	27 (0.6%)	30 (0.5%)	83 (0.5%)
**Investigator decision**	13 (0.3%)	1 (0.0%)	3 (0.0%)	17 (0.1%)
**Other**	212 (4.1%)	294 (6.4%)	394 (6.1%)	900 (5.5%)
**Refused further participation**	948 (18.3%)	1103 (24.1%)	1639 (25.3%)	3690 (22.7%)
**Relocated, no follow-up planned**	3500 (67.4%)	2970 (64.8%)	4240 (65.4%)	10,710 (65.9%)

^a^PC36 visit status was reclassified as “Missed visit” if the withdrawal reason was “unable to contact participant”

In univariable analyses, nearly all variables were significantly associated with retention (*p* < 0.05), except for employment status, self-reported every being told they had tuberculosis, and nights away from home (Table 4). The final multivariable model included the following variables: sex, age, education, employment, marital status, SES level, study arm, baseline HIV status, self-reported HIV status/care, AUDIT risk level, recreational drug use, multiple sex partners in the past 12 months, and condom-less last sex, while adjusting for study triplet (regression output not shown). Adjusted RRs were largely consistent with univariable results for sex, age, education, marital status, SES level, and study arm (Table 4). Retention was lower in men compared to women (adjusted (adj) RR = 0.90, 95% CI: 0.88, 0.91), increased with age (adj RR for 25–34 to 18–24 = 1.08, 95% CI: 1.06, 1.10; adj RR for 35–44 to 18–24 = 1.23, 95% CI: 1.20, 1.26). Those with highest educational attainment tended to have lower retention (adj RR for college/university education compared with none to primary school = 0.90, 95% CI: 0.87, 0.94), whereas rates were similar for those with full to partial secondary school, when compared with none to primary school (adj RR = 1.00, 95% CI: 0.97, 1.02). Employment status was significant in the multivariable model, with highest retention in students (adj RR comparing to unemployed = 1.07, 95% CI: 1.04, 1.10) and similar retention in the employed and unemployed groups (adj RR comparing employed to unemployed = 1.02, 95% CI: 1.00, 1.04). Participants who were married had highest retention, followed by formerly married (adj RR for formerly married to married = 0.99, 95% CI: 0.96, 1.03), and never married having lowest retention (adj RR for never married to married = 0.96, 95% CI: 0.94, 0.98). Retention was higher among those with higher SES level (adj RR for med to low SES = 1.12, 95% CI: 1.09, 1.14; adj RR for high to low SES = 1.16, 95% CI: 1.14, 1.19). By arm, retention was lowest in arm A, followed by arm C (adj RR compared to arm A = 1.02, 95% CI: 0.99, 1.04) and highest in arm B (adj RR compared to arm A = 1.04, 95% CI: 1.02, 1.06). For self-reported HIV status, HIV-positive status showed increased retention (adj RR comparing positive/registered for care to self-reported HIV-negative = 1.16, 95% CI: 1.12, 1.21; adj RR comparing positive/never registered for care to self-reported HIV-negative = 1.08, 95% CI: 1.02, 1.15). In contrast, participants with an HIV-positive laboratory test result were less likely to be retained compared to HIV-negative individuals, (adj RR = 0.85, 95% CI: 0.83, 0.88). AUDIT score, recreational drug use in the past 12 months, and multiple sex partners were not significantly associated with retention in the multivariable model.

**Table 4:** Univariable and multivariable analysis of study retention rate

	**Total retained visits**	**Univariable**		**Multivariable**	
**Level**	**Mean (SE)**	**Rate ratio (95%CI)**	***P*-value**	**Adjusted RR (95% CI)**	***P*-value**
Sex (Ref = female)	1.82 (0.01)	1	< .001	1	< .001
Male	1.61 (0.01)	0.88 (0.87, 0.90)		0.90 (0.88, 0.91)	
Age (Ref = 18–24)	1.64 (0.01)	1	< .001	1	< .001
25–34	1.74 (0.01)	1.06 (1.04, 1.08)		1.08 (1.06, 1.10)	
35 +	2.00 (0.01)	1.22 (1.19, 1.24)		1.23 (1.20, 1.26)	
Education (Ref = none to primary school)	1.76 (0.02)	1	0.031	1	< .001
Full to partial secondary school	1.77 (0.01)	1.00 (0.98, 1.02)		1.00 (0.97, 1.02)	
College/university	1.69 (0.03)	0.96 (0.93, 1.00)		0.90 (0.87, 0.94)	
Employment (Ref = unemployed)	1.75 (0.01)	1	0.096	1	< .001
Student	1.75 (0.02)	1.00 (0.98, 1.02)		1.07 (1.04, 1.10)	
Employed	1.78 (0.01)	1.02 (1.00, 1.04)		1.02 (1.00, 1.04)	
Marital Status (Ref = married/living as married)	1.83 (0.01)	1	< .001	1	0.002
Divorced/separated/widowed	1.80 (0.02)	0.98 (0.96, 1.01)		0.99 (0.96, 1.03)	
Never married	1.70 (0.01)	0.93 (0.91, 0.94)		0.96 (0.94, 0.98)	
Country-specific SES (Ref = low)	1.63 (0.01)	1	< .001	1	< .001
Medium	1.80 (0.01)	1.10 (1.08, 1.13)		1.12 (1.09, 1.14)	
High	1.89 (0.01)	1.16 (1.14, 1.18)		1.16 (1.14, 1.19)	
Arm (Ref = Arm A)	1.72 (0.01)	1	0.008	1	0.008
Arm B	1.78 (0.01)	1.04 (1.02, 1.05)		1.04 (1.02, 1.06)	
Arm C	1.75 (0.01)	1.02 (1.00, 1.04)		1.02 (0.99, 1.04)	
Baseline HIV status (lab confirmed) (Ref = Negative)*	1.79 (0.01)	1	< .001	1	< .001
Positive	1.69 (0.01)	0.95 (0.93, 0.97)		0.85 (0.83, 0.88)	
Self-report HIV status and care (Ref = negative)	1.77 (0.01)	1	< .001	1	< .001
Don’t know	1.73 (0.01)	0.98 (0.96, 1.00)		0.97 (0.96, 0.99)	
Never tested	1.68 (0.02)	0.95 (0.93, 0.97)		0.96 (0.93, 0.98)	
Positive, have registered for care	1.91 (0.02)	1.08 (1.05, 1.11)		1.16 (1.12, 1.21)	
Positive, never registered for care	1.76 (0.04)	0.99 (0.95, 1.04)		1.08 (1.02, 1.15)	
Ever told you have tuberculosis (Ref = no)	1.76 (0.01)	1	0.054		
Yes	1.86 (0.06)	1.06 (1.01, 1.13)			
AUDIT risk level (Ref = low risk)	1.76 (0.01)	1	0.002	1	0.144
Medium risk	1.68 (0.03)	0.96 (0.93, 0.99)		0.99 (0.96, 1.02)	
High risk	1.67 (0.04)	0.95 (0.91, 1.00)		1.05 (1.00, 1.11)	
Days off sick from work (past 3 mo.) (Ref = none)	1.75 (0.01)	1	0.204		
1 to 5	1.80 (0.03)	1.03 (0.99, 1.06)			
> 5	1.73 (0.03)	0.98 (0.95, 1.02)			
Nights away from home (past 3 mo.) (Ref = none)	1.74 (0.01)	1	0.062		
1 to 7	1.81 (0.02)	1.04 (1.01, 1.06)			
8 to 30	1.76 (0.04)	1.01 (0.97, 1.05)			
> 30	1.78 (0.07)	1.02 (0.94, 1.10)			
Used drugs recreationally (past 12 mo.) (Ref = No)	1.76 (0.01)	1	< .001	1	0.285
Yes	1.62 (0.04)	0.92 (0.88, 0.96)		0.97 (0.92, 1.03)	
Multiple sex partners (past 12 mo.) (Ref = no)	1.77 (0.01)	1	< .001	1	0.929
Yes	1.63 (0.03)	0.92 (0.89, 0.95)		1.00 (0.97, 1.04)	
Condom-less last sex (Ref = no)	1.74 (0.01)	1	0.018	1	0.049
Yes	1.78 (0.01)	1.02 (1.00, 1.03)		0.98 (0.96, 1.00)	

^*^The following variables were selected for inclusion in the multivariable analysis based on *P* < 0.05 on univariable analysis: sex, age, education, employment, marital status, country-specific SES, arm, baseline HIV status (lab confirmed), self-reported HIV status and care, AUDIT risk level, used drugs recreationally, multiple sex partners, condom-less last sex

## Discussion

Of participants enrolled at PC0, 73% had at least one follow-up visit completed (Table 2). Baseline characteristics were overall balanced across study arms, with the exception of more participants with higher SES in arm C. Arm B participants were marginally more likely to be retained compared to arms A and C. In keeping with previous studies of varied types and locations, retention was lower among men and younger participants (18–24 years) [4, 9–11], and higher among participants resident in higher SES households [9]. In contrast to previous studies, behavioral risk factors including multiple sexual partners and alcohol and drug use showed no association with retention [11].

Retention in PopART was lower than that achieved in the Botswana Combination Prevention Project (BCPP). BCPP was a smaller CRT, conducted in a more rural setting (15 villages in Botswana) between 2013 and 2018 that evaluated the effect of enhanced provision of ART and voluntary male medical circumcision on population level HIV incidence. The CRT design in PopART and BCPP was comparable with study interventions provided community-wide and a cohort of community members randomly selected for more intensive follow-up for primary end point assessment. Study interventions were context specific and similar to PopART. In BCPP, 12,600 participants were followed up for HIV testing at 12 and approximately 29 months. Overall, > 95% of participants enrolled into the study cohort were successfully followed up at either 12 or 29 months [18]. No formal comparison of retention has been made across the two studies; however, the smaller number of participants in BCPP perhaps facilitated more intensive follow-up and thus higher retention. The more rural setting in which BCPP took place, perhaps with more social cohesion and less in and out migration of people compared to the peri-urban communities included in PopART, may also have impacted retention; however, further in-depth comparisons are needed to properly understand these differences [18, 19].

The association between HIV status and retention produced some interesting findings. Participants confirmed as HIV positive on baseline laboratory testing were significantly less likely to be retained. Far fewer participants self-reported HIV positive status at baseline than those confirmed on laboratory HIV testing. Participants self-reporting HIV positive at baseline were conversely more likely to be retained compared to those reporting “not knowing their status” or “never having tested.” The difference in frequency of HIV positive status between laboratory and self-report is likely to reflect a combination of “not knowing HIV status” and under-reporting of known HIV-positive status by participants, a trend supported by previously published data from the PC which showed that a considerable number of participants who were on ART self-reported they were HIV negative [20]. Non-disclosure of HIV status reflecting social desirability [21] and linked to stigma in a range of contexts [22] is well reported and likely to have contributed to under-reporting, especially given issues around confidentiality with surveys being completed inside participant households. Participants who did report an HIV-positive status, especially those in HIV care, were more likely to be retained in PopART, in keeping with a reported positive association between knowledge and disclosure of HIV status and improved adherence to HIV-related healthcare [23].

Among participants defined as having permanently left the study (study “withdrawal”), the most common reason documented by the research team was “moving out of the study catchment” area (66%). This was despite “intention to remain in community over the 3-year period of study activities” being a study eligibility criterion. This suggests that participants who exited the community during the trial period were likely to have done so without prior long-term planning. In a separate PopART publication, social cohesion within study communities as the result of complex community dynamics (such as availability of social amenities, crime, poverty and drug use) was related to migration in and out of study communities. Where information is available, these factors should be considered in the choice of study communities and once the study is implemented a better understanding of these issues could assist to focus retention interventions [24].

There has been extensive research into the effectiveness of retention strategies in longitudinal studies. A systematic review of 141 cohort studies from 28 countries [25] categorized retention strategies as (i) barrier-reduction, (ii) creating a project community, and (iii) follow-up/reminder strategies. “Barrier reduction” interventions, aimed at assisting participants to attend study visits, e.g., provision of transport to research sites, were found to be the most effective in improving retention outcomes. Individuals with lower SES were found to experience increased barriers toward research participation, including financial barriers, language barriers, transport access barriers, low health literacy levels, lack of awareness of research trials, and distrust of healthcare systems [26], all of which may also be applicable to community-based CRTs, and this is supported by our study finding of better retention among individuals of higher SES status [9].

The need for disclosure by participants about study participation and support for participants within their households and communities is recognized as critical for retention [6, 27], as is wider support within trial communities [27]. Similar to that for ART program implementation [28], there is increasing consensus of the need to embrace a client-centered approach inclusive of extensive community engagement and partnership for successful completion of clinic- and community-based longitudinal studies [29, 30]. Within this framework of community partnership, a better understanding of specific factors associated with retention can enable interventions with an enhanced focus on participant groups more likely to experience retention losses, such as men and younger age groups [4, 9, 31].

PopART has provided a unique opportunity to evaluate participant retention in the largest HIV prevention CRT to date. The community-based follow-up, over a long period of time (3–4 years), with inclusion of urban and peri-urban areas is also novel and contributes to the scientific relevance of this study. Standardized retention activities with active participant follow-up and careful documentation of these efforts, together with publications communicating associated outcomes from the study, have enabled us to complete a rigorous evaluation of participant retention. However, limitations for consideration exist. An approximately equal number of men and women were randomly selected for study enrolment. Men, however, were harder to access as they were often not present in the house and had higher rates of refusal to participate in the study. We unfortunately do not have systematic data on participants who refused to participate in the study. Men were more likely to be lost to follow-up and having more women in the cohort may therefore have increased overall retention. Similarly, reporter bias and “social desirability” among participants may have led to under-reporting of social activities such as alcohol and drug use which in turn affected the multivariable analysis. Conversely with the very large sample size, there becomes a high likelihood of statistically significant associations between independent variables and study outcomes even when differences in the proportions with the outcome are small across variable categories, and this should be considered when reviewing the multivariable analysis. Although the 21 communities in Zambia and South Africa included in PopART are representative of many high HIV burden communities, contextual factors (e.g., urban vs. peri-urban) likely play a significant role in determining participant retention. The findings of this study therefore need to be reviewed carefully when conducting similar studies in other settings. Our primary retention outcome was defined as the total number of annual visits where a study questionnaire was completed. For the participant visit to contribute to primary endpoint measurement laboratory-based HIV testing needed to be completed. There were a limited number of retained participants for whom no laboratory-based HIV testing was done for a variety of reasons including failure to complete phlebotomy and hemolyzed samples. Evaluation of this HIV testing pathway was outside the focus of this paper; however, challenges with testing should also be carefully considered by researchers implementing community-based trials.

## Conclusions

Effective strengthening and innovation of disease programs in high HIV burden settings are reliant on high-quality evidence, often from longitudinal studies including CRTs. Retention in CRTs remains a major challenge. In this study, we confirmed lower retention among men, youth, and those with low SES, highlighting the need to consider focussed strategies to retain groups at high risk of loss to follow-up. Movement within and between study communities is a significant challenge for CRTs and anecdotal reports from PopART highlight the need to be mindful of this during study design. The PopART study invested significantly in the development of stakeholder relationships during study implementation and working closely with communities to implement community-based trials should be a priority.

## Data Availability

Data used in this study will be available on request immediately after publication, with no end date. This includes de-identified participant data with a data dictionary. A data archive will be held at Fred Hutchinson Cancer Center, Seattle, WA, USA. Requests can be sent to HPTN-Data-Access@scharp.org.
